# Blockchain Integration With Digital Technology and the Future of Health Care Ecosystems: Systematic Review

**DOI:** 10.2196/19846

**Published:** 2021-11-02

**Authors:** Hanaa Fatoum, Sam Hanna, John D Halamka, Douglas C Sicker, Peter Spangenberg, Shahrukh K Hashmi

**Affiliations:** 1 College of Medicine Alfaisal University Riyadh Saudi Arabia; 2 School of Professional & Extended Studies American University Washington, DC United States; 3 Mayo Clinic Platform, Mayo Clinic Rochester, MN United States; 4 School of Computer Science Carnegie Mellon University Pittsburgh, PA United States; 5 King Faisal Specialist Hospital and Research Center Riyadh Saudi Arabia; 6 Division of Hematology Mayo Clinic Rochester, MN United States

**Keywords:** blockchain, Internet of Things, digital, artificial intelligence, machine learning, eHealth, ledger, distributed ledger technology

## Abstract

**Background:**

In the era of big data, artificial intelligence (AI), and the Internet of Things (IoT), digital data have become essential for our everyday functioning and in health care services. The sensitive nature of health care data presents several crucial issues such as privacy, security, interoperability, and reliability that must be addressed in any health care data management system. However, most of the current health care systems are still facing major obstacles and are lacking in some of these areas. This is where decentralized, secure, and scalable databases, most notably blockchains, play critical roles in addressing these requirements without compromising security, thereby attracting considerable interest within the health care community. A blockchain can be maintained and widely distributed using a large network of nodes, mostly computers, each of which stores a full replica of the data. A blockchain protocol is a set of predefined rules or procedures that govern how the nodes interact with the network, view, verify, and add data to the ledger.

**Objective:**

In this article, we aim to explore blockchain technology, its framework, current applications, and integration with other innovations, as well as opportunities in diverse areas of health care and clinical research, in addition to clarifying its future impact on the health care ecosystem. We also elucidate 2 case studies to instantiate the potential role of blockchains in health care.

**Methods:**

To identify related existing work, terms based on Medical Subject Headings were used. We included studies focusing mainly on health care and clinical research and developed a functional framework for implementation and testing with data. The literature sources for this systematic review were PubMed, Medline, and the Cochrane library, in addition to a preliminary search of IEEE Xplore.

**Results:**

The included studies demonstrated multiple framework designs and various implementations in health care including chronic disease diagnosis, management, monitoring, and evaluation. We found that blockchains exhibit many promising applications in clinical trial management such as smart-contract application, participant-controlled data access, trustless protocols, and data validity. Electronic health records (EHRs), patient-centered interoperability, remote patient monitoring, and clinical trial data management were found to be major areas for blockchain usage, which can become a key catalyst for health care innovations.

**Conclusions:**

The potential benefits of blockchains are limitless; however, concrete data on long-term clinical outcomes based on blockchains powered and supplemented by AI and IoT are yet to be obtained. Nonetheless, implementing blockchains as a novel way to integrate EHRs nationwide and manage common clinical problems in an algorithmic fashion has the potential for improving patient outcomes, health care experiences, as well as the overall health and well-being of individuals.

## Introduction

The blockchain concept was first described 3 decades ago and was meant to be used as a digital timestamp for documents to prevent tampering, functioning somewhat like a notary. However, it did not develop significantly and went largely unnoticed until the global financial crisis. In 2008, the blockchain revolution began when Nakamoto pioneered and crystallized it by releasing his whitepaper [1] followed by his cryptocurrency called Bitcoin, offered as an open-access protocol to the public.

Blockchain is considered one of today’s important ground-breaking technologies. The question is what makes blockchain so unique and useful.

In simple words, it provides digital trust and transparency, something that has not only been seriously lacking in the digital world but is also posing major challenges in an era of increased dependence on electronic data, along with viewing the shift toward digitization and substituting other traditional methods of data storage as a glorified goal. A caveat that needs to be considered is that many digital health start-ups have failed, given their inability to convince the investors and users or because of using older technologies that become outdated by the time a completely digital health system is established.

The security of digital data, and the fact that it can be manipulated, tampered with, and purposefully hidden to suit the parties of interest, can be quite an alarming thought. Security concerns have led to resistance toward the use of electronic cloud-based data. However, using blockchain technology can potentially provide a breakthrough.

We will evaluate cases involving 2 patients in typical yet different clinical scenarios and then analyze the role of blockchains in the management of these patients.

Chenoa is an 8-year-old girl from Cheyenne, Wyoming, who was recently diagnosed with acute lymphoblastic leukemia. Her parents were farmers and there was no large tertiary care center in town that could provide treatment for life-threatening cancers with aggressive chemotherapy. They traveled to Colorado to obtain specialized care as recommended by their local hematologist. She received all her initial treatments in Denver, followed by a stem cell transplant from her elder brother as the donor after receiving massive radiation and chemotherapy for the transplant. Moreover, 4 months posttransplant, there was no evidence of leukemia or rejection (graft-versus-host disease), and they return to Cheyenne to celebrate their cancer conquest with the rest of the Cherokee tribe. A month after returning, Chenoa develops high-grade fever and is diagnosed with an extremely low white blood cell count along with a relapse of her leukemia at the local hospital. Immediate transfer to the transplant center in Colorado is recommended.

Khaled is a 58-year-old retired banker and an ex-smoker who lives in Dearborn, Michigan, with hypertension, coronary heart disease, and chronic kidney disease for which he undergoes hemodialysis thrice a week and is on the renal transplant list for a transplant. He is divorced and single; however, his daughter, who lives in Cleveland (Ohio), frequently visits him. He was recently diagnosed with heart failure; given the precarious health system and lack of caretakers in Dearborn, he is temporarily planning to move to his daughter’s house.

The above 2 examples characterize the real-world scenarios within the United States, a developed country; despite incurring some of the highest health care costs, the United States has a disjointed health care system as far as digital health is concerned. Given the impediments and complexities in the US health care system, errors and wastage within the health ecosystem can directly affect patient outcomes like those of Chenoa and Khaled.

Chenoa’s transfer was delayed given that she became increasingly unstable, as her infection led to septic shock. Though intensive care unit (ICU) management was optimum, and she was receiving multiple antibiotics and vasopressors, hopes for her stabilization and transfer to Denver were diminishing. A couple of days prior to hospital admission, Chenoa was taken to another local hospital because of fever and flu-like symptoms, where she was given an outpatient prescription of antihistamines. Though her transplant team had instructed the parents to telephone them for any issues, this was a very minor issue, and not entirely unexpectedly, the local physician in the community did not realize the depth of the immunosuppressive state that Chenoa exhibited. A week after highly aggressive treatment in the ICU, Chenoa died of multiple organ failure due to sepsis.

Khaled’s story had a different twist; his daughter made a hasty decision to take him to Ohio, but he deteriorated quickly. She had to take him directly to a large tertiary care center in Cleveland where the emergency room doctors evaluated and triaged him appropriately and were relentlessly trying to obtain medical records from Detroit where Khaled’s doctors were located. He went into cardiac arrest twice within a few hours of arrival and cardiopulmonary resuscitation was stopped after a prolonged effort following discussions with his daughter, as it seemed futile.

Fortunately, most of the patients in the United States do not exemplify the above cases; however, similar issues routinely occur given the lack of trustworthy and secure digital infrastructure for health care. Many questions arise after the death of Chenoa and Khaled, the most essential of which is whether there was a medical error on the side of the individuals or the health care ecosystem, which led to their fatal demise ( eg, were these deaths preventable?).

We provide a systematic review of literature on the novel health care ecosystem focusing on blockchains and then consider the cases of Chenoa and Khaled based on the current data.

## Methods

### Literature Search

**Figure 1:**
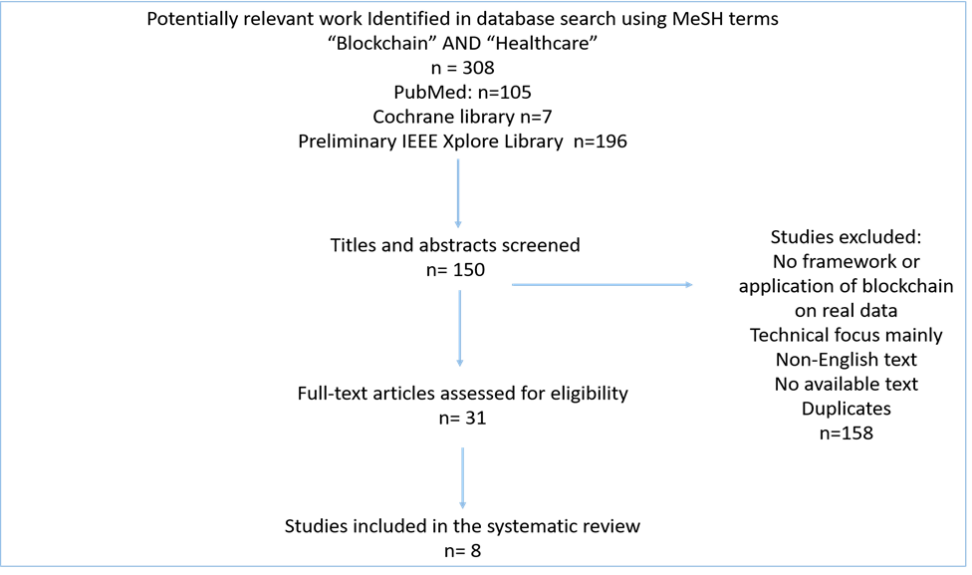
Systematic review outcome. MeSH: Medical Subject Headings, IEEE: Institute of Electrical and Electronics Engineers.

The authors searched through the literature using the terms “blockchain” and “healthcare” based on Medical Subject Headings to identify related existing work. The inclusion criteria were focused on studies related to health care and clinical research; a working framework was proposed as well as implementation and testing with data. Exclusion criteria are presented in Figure 1*.*

The literature sources for this systematic review were PubMed, Medline, and the Cochrane library in addition to a preliminary search of IEEE Xplore, which had works with a technical focus rather than a health care outcome focus.

Reviewers created a data extraction sheet and identified the required data, and 31 full-text studies were critically evaluated; among these, 9 studies were finally selected, and they are summarized in Table 1.

First, we provide an overview of the essential elements of blockchains for readers and then explain the specific methodology in detail.

### Overview of Blockchain Technology

The 3 pillars of blockchain technology that make it a revolutionary technology are decentralization, transparency, and immutability. A block is a virtual data storage unit that holds records, transactions, or other means of data. Each block is chronologically linked to the previous blocks using cryptography; once a block is created, it is permanently stored on the blockchain and cannot be modified or removed, thus becoming immutable. If one wishes to update a network node, make another transaction, or add new data, a new block must be added to the chain; all the previous blocks will still be unchanged and visible. Another aspect of a blockchain is that it is a decentralized database using the distributed ledger technology, which simultaneously stores a full replica of the data on multiple nodes, unlike most other data management technologies where data storage is centralized, meaning that it is stored at a single location, mostly on a single server or mainframe computer [2].

The immutability of a blockchain is attributed to different factors; each block has an autogenerated header, which has a unique identification and a timestamp. The block also has a hash key, which is the header of the previous block. Therefore, all blocks are interlocked within their respective blockchains.

Once a verified permissioned node (node C) wants to add new data or make a transaction, a request is sent to all associated blockchain nodes to verify that the content of node C’s blockchain matches the content of all the other nodes (nodes A, B, etc). In addition to ensuring a complete match of all the block headers, a unique signature header will be generated for the new block. The new block is added to the blockchain only after the approval of the node.

Blockchains may be permissionless (public), permissioned (private), or sometimes hybrid, as described below. Public blockchains allow any user to join, view, and add data to the ledger, which offers maximal transparency. However, private blockchains allow only those with access to interact with the network and are usually controlled and maintained by a single organization offering superior privacy and scalability compared to public blockchains. Finally, in consortium-based blockchains, which are hybrids of the other 2 types, the configuration for viewing and writing access is determined by a group of organizations or entities.

Based on this general overview of blockchain technology, we present the salient features of blockchains, which include cybersecurity, applications, and various domains associated with them.

## Results

The results of this systematic review alluded to a wide variety of applications for blockchains and various technical differences in the methods used to establish a blockchain [3-10]; a summary is provided in Table 1*.*

All the studies pertained to electronic health records (EHRs), clinical trials [6-8], or device integration using Internet of Things (IoT) [3,5,9,10].

Researchers at the Chinese Institute of Physical Science have proposed a model called Med-PPPHIS, which combines permissionless and permissioned blockchains, aiming at a closed-loop method for chronic disease management; the authors used Med-PPPHIS for national physique monitoring and scientific exercise guidance using various self-invented IoT medical devices such as health parameter assessment tools, athletic and functional performance assessment devices, wearable heart rate monitors, and intelligent fitness equipment. The blockchain was tested by 25 virtual machine simulations over 500 nodes, with results revealing superior security, higher data transmission rates, and low latency [3]. Another method was used by Hylock and Zeng et al, in which they used a proof-of-concept tool to extensively test all the 16 configurations of their proposed framework in a variety of scenarios, and they demonstrated results similar to the above study [4].

The blockchain was integrated with IoT devices for evaluating and monitoring essential tremor disease; herein, patients were able to use their smartphones to report their location and activity, self-evaluate their disease activity, and log aggravating and relieving factors, in addition to the data from their smartwatches and multiple air systems, providing a holistic view of their disease status. The authors concluded that blockchains resulted in increased efficiency, scalability, decreased cost, and flexibility in data access management [5]. Other studies have employed IoT devices and blockchains for patient monitoring [9,10]. They have created systems that can analyze and manage medical sensor data as well as send alerts based on patient-customized threshold values and abnormal patterns using smart contracts while simultaneously integrating the data into the patients’ EHRs.

The last 3 studies [6-8] focused more on the application of blockchains in biomedical research and clinical trials providing proof-of-concept frameworks featuring customized smart contracts, which allowed for more control over data access depending on researcher privilege levels and patient-controlled authorization. Researchers have also incorporated additional security measures such as biometric verification for physician access. Additionally, blockchains have been used as a solution for continuous trial monitoring by clinics, financial sponsors, and participants.

Blockchains are instrumental in many clinical and research domains. Therefore, some governments are exploring the option of having a nationwide blockchain for the EHRs of all their citizens. Estonia is the first country in the world with a digital health care ecosystem for EHRs based on blockchain technology and has provided the world with a model for data integrity and efficiency. Some other countries are in the process of adopting blockchains at the macroscopic level to reduce health care waste, increase efficiency, and ultimately improve outcomes. However, besides the conventional risks of technological failure, scalability remains a challenge; for instance, conducting 800 transactions per second for hyperledgers using blockchains to store continuous telemetry data is not yet practical. Nonetheless, researchers hope that with the current advances in technology, specific solutions for storage optimization and redesigning of blockchain will be available soon [11]. One additional study [12] evaluated the performance of a blockchain-based online machine learning tool available on the internet called ExplorerChain, which uses 3 separate and different data sets (myocardial infarction, cancer biomarkers, and length of hospitalization). The study concluded that the performance of ExplorerChain was as good as the central server–based algorithm while providing the benefits of a distributed model. Nevertheless, the tradeoff with some of those benefits was the cost of efficiency. However, with the rise of supercomputers, the costs associated with running a blockchain are likely to decrease over time.

Let us return to the unfortunate cases of Khaled and Chenoa. Imagine that they lived in a digitalized nation (ie, a smart country as opposed to a smart city), where all the EHRs were on a private or a consortium-based blockchain.

**Table 1:** Summary of data extraction results.

Reference	Title	Platform or model	Implementation	Features	Method (blockchain interface and IoT^a^ device)
Zhou et al [3]	Med-PPPHIS: blockchain-based personal healthcare information system for national physique monitoring and scientific exercise guiding	Med-PPPHIS and Med-DLattice blockchain	Chronic disease management: physique monitoring	Chronic disease management target; scientific and personalized exercise prescriptions (electronic prescriptions), providing users with safe, effective, and private scientific health guidance for the management of chronic diseases	Web portal and mostly self-developed medical IoT devices such as health sign monitoring equipment, heart rate monitor, and intelligent fitness equipment
Hylock and Zeng [4]	A blockchain framework for patient-centered health records and exchange (HealthChain): evaluation and proof-of-concept study	HealthChain	Patient-centered blockchain framework	Smart contracts, proxy re-encryption, revocable access, and patient-centered framework	Web portal
Zheng et al [5]	Accelerating health data sharing: a solution based on the Internet of Things and distributed ledger technologies	IOTA tangle	Remote diagnosis of essential tremor disease	App allowing users to report their location, activity, and tremor level; self-evaluation of the disease and other factors related to the disease, such as medication and alcohol consumption	App; wearable devices (Pebble smartwatch) and stationary air quality sensors
Zhuang et al [6]	Applying blockchain technology for health information exchange and persistent monitoring for clinical trials		Patient-reported outcomes for trials; EHR^b^ sharing	Private blockchain, smart contracts, biometric verification of physicians, and patient-controlled data access	Web portal
Johnson et al [7]	Building a secure biomedical data sharing decentralized app (DApp): tutorial	Oasis Devnet/Ethereum	Biomedical research	Smart contracts, public code for app recreation, and geolocation sharing	iPhone (iOS) app
Maslove et al [8]	Using blockchain technology to manage clinical trials data: a proof-of-concept study	BlockTrial/ Ethereum protocol	Clinical trials	Smart contracts and mediated data access based on patient-granted permissions	Web app
Satamraju and Malarkodi [9]	Proof of concept of scalable integration of Internet of Things and blockchain in healthcare	DApp/Ethereum protocol	Remote patient monitoring	Smart contracts, off-chain storage, and Ethereum-based system users including patients, doctors, pharmacists, and insurance companies	App; pulse oximetry device, body temperature sensor, and room temperature sensor
Griggs et al [10]	Healthcare blockchain system using smart contracts for secure automated remote patient monitoring	DApp /Ethereum protocol	Automated patient monitoring	Smart contracts, custom threshold values for alerts, Oracle (master device to control smart contacts), and integration with EHRs	App; medical IoT devices
Kuo et al [11]	Expectation propagation logistic regression on permissioned blockchain (ExplorerChain): decentralized online healthcare/genomics predictive model learning	ExplorerChain	Blockchain combined with artificial intelligence for health care and genomics; predictive model training	iDASH private HIPAA^c^-compliant computing environment network; applied on 3 different data sets including myocardial infarction, cancer biomarkers, and length of hospitalization	Distributed servers using internet-based machine learning

^a^IoT: Internet of Things.

^b^EHR: electronic health record.

^c^HIPAA: The Health Insurance Portability and Accountability Act of 1996.

## Discussion

### Cybersecurity

EHRs play a vital role in providing smooth, safe, and efficient health care delivery. As many countries do not currently possess a unified health care system, data sharing and interoperability (in a secure manner) become essential for providing patients with the best care. However, this has been a major issue in current health care management systems. Data are one of the most valuable commodities; since the increased reliance on digital systems, cybercriminals have adjusted their methods owing to huge financial incentives, especially through selling the identifying data of people. Thus, medical records are currently worth more than social security numbers on the black market, as they include the date of birth, home addresses, contact data, health data, and other sensitive data. EHR data can be employed for various criminal activities like identity frauds, insurance frauds, phishing, and ransomware, leaving the patients compromised and susceptible to harm [13]. Cyberattacks on hospital systems have increased worldwide in the past decade; such attacks not only impede health care delivery and cause financial losses but also affect patients' trust in medical providers [13,14].

In 2018, a cyberattack on SingHealth (Singapore Health Services) compromised the records of 1.5 million patients [15]; it is considered one of the biggest data breaches in Singapore and worldwide. It has stirred considerable controversy about the security of patients’ data and the reasons behind not implementing any significant measures for changing the EHR data systems. Compared to the conventional methods of EHR storage, a blockchain is a potentially secure and an immutable method for data storage and management owing to its decentralized nature. This means that the latest version of the chain is replicated, sent, and widely distributed in a huge network of nodes; there are no weak links for hackers to breach. Each block has a key of its own, in addition to having the hash key of the previous block. Once a transaction request has been made by a user, the blockchain protocol requests the network nodes to verify the validity of the entire blockchain content. This in turn means that unless the networks nodes verify that the current version of the chain is identical to theirs and approve the transaction, it cannot be added to the chain [16,17].

Furthermore, the transaction validation uses cryptominers, which are nodes that possess specialized hardware and software capable of solving energy-intensive cryptographic puzzles. It would be extremely difficult for someone to gather enough computing power to hack the blockchain database by altering the ledgers. The larger the blockchain, the more distributed it is, the more enormous is the computing power required for hacking, and the more secure it becomes [16,17].

Finally, digital signatures are employed to verify the identity of those who wish to access or add data to the blockchain. Additional features such as hardware security modules (HSMs) can be added as an additional layer to further enhance the protection of the patients’ data. HSMs are specialized hardware devices that are used to guard highly sensitive data. An HSM acts as a trusted network node that performs several cryptographic processes such as key generation and management, as well as encryption and decryption of digital signatures. HSMs are usually placed in a secure physical location and cannot be accessed, thereby making them highly tamper-resistant systems [18].

### Applications and Domains of Use in Health Care

Blockchain technology has thrived in many industries ranging from banking to supply chain management. It is predicted to have a major impact on the health care industry. According to the forecast report for 2018 to 2023 provided by Market Research Future, the global blockchain market is predicted to expand exponentially.

Fundamentally, from a patient’s perspective, the potential role of a blockchain in developing a patient’s personal health record could be significant. The patient-facing applications of this technology would benefit from one window and one operating system for the personal health records, out-of-pocket costs, covered versus uncovered services, clinical trial searches, consenting for clinical trials, and “omics” data interpretation.

**Figure 2:**
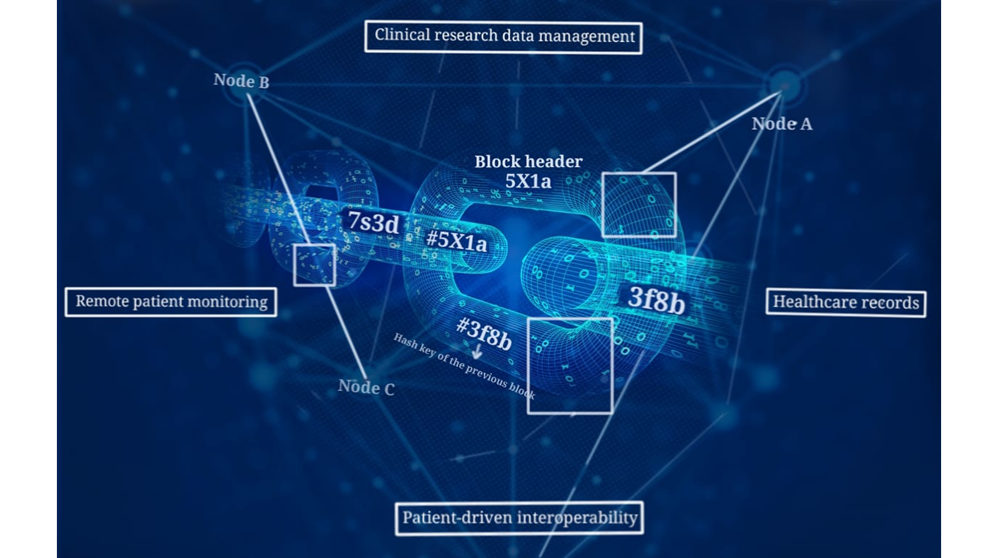
Illustration of blockchain usage in health care.

**Figure 3:**
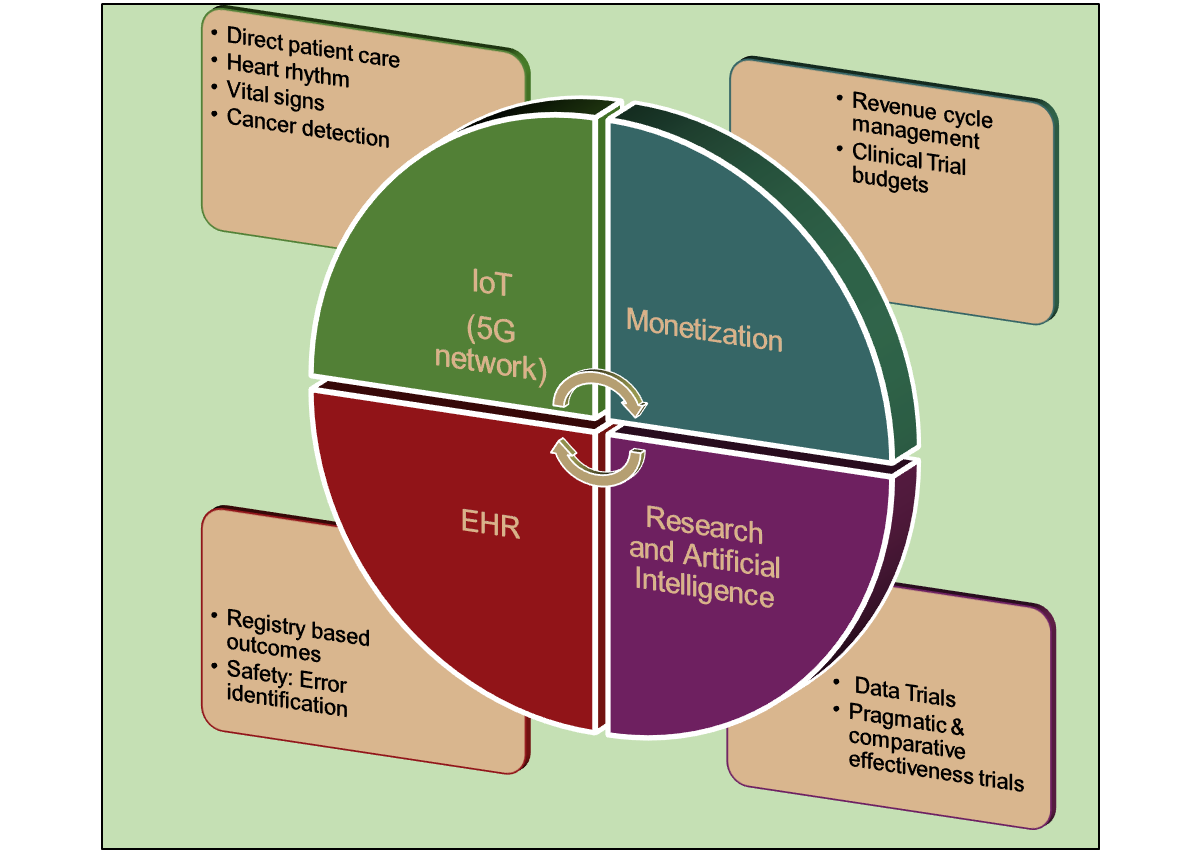
Areas of Blockchain utilization in healthcare.

The review focuses on 5 main areas in which most health care–related implementations fall under remote patient monitoring (including IoT devices), EHRs, patient-centered interoperability, clinical trial data management, and monetization, as shown in Table 2 and Figure 2. The potential application areas of blockchain technology in health care are depicted in Figure 3.

**Table 2:** Areas of blockchain implementation.

Application area	Blockchain technology features
Electronic health record management	Patient-reported outcomes Consent
Patient-centered interoperability	DNR^a^ orders Instantaneous data access and interoperability
Remote patient monitoring	Patient-mediated and controlled record access IOT^b^-enabled monitoring of vital signs, glucose, and other parameters Disease surveillance and outbreak management
Clinical trial data management	Increased RCT^c^ data transparency and quality IOT-generated clinical research data Smart contracts applying data specifications and incentives
Monetization	Revenue cycle management Clinical trial budgets

^a^DNR: do not resuscitate.

^b^IoT: Internet of Things.

^c^RCT: randomized control trial.

### The IoT Concept, Blockchain, and Clinical Trials

The integration of blockchains with other technologies such as artificial intelligence (AI), IoT, and big data management can be highly effective and act as a catalyst for innovation and increased efficiency, which is invaluable for the interpretation and management of data.

IoT refers to a world where people, things, and devices are all connected through the internet, allowing them to collect and exchange data seamlessly over the network. From coffee machines, wearable devices, and sensors to home security systems, IoT will likely change the way people live because 5G wireless technology (with high bandwidth and low latency) is becoming readily available. All the data streamed over the network can interlock without the need for human interactions. Future smart cities are based on IoT devices and applications. Additionally, it has been gaining considerable attention from stakeholders, investors, and various organizations owing to its unlimited application possibilities [19-21].

Data from sensors and wearable devices can revolutionize the way health care is viewed and delivered, especially in an era of patient-centered care, precision medicine, and individualized health care delivery. IoT can transform the approach to health care and take patient-centered care to a new level, where people can take charge of their health, providing patients and physicians with invaluable continuous real-time data about the physiological state and well-being of patients, ranging from data such as the heart rate, temperature, or sleeping habits to biochemical marker measurements in biofluids through various biosensor technologies, as well as sending alerts when certain thresholds are crossed or abnormal patterns are detected [22-27].

Using IoT technology in conjunction with blockchain technology can maximize its efficacy and potential uses. The massive amounts of various data streamed through different IoT devices can be used to collect large amounts of invaluable data for researchers to analyze and interpret. It could also be useful for data-hungry AI technology companies, public health surveillance, monitoring of disease outbreaks (eg, for monitoring COVID-19), epidemiology, and patient-oriented outcomes [28-33].

AI has been one of the key catalysts in health care innovation; for instance, researchers at the Massachusetts Institute of Technology made a ground-breaking discovery of a new antibiotic using AI technology through a trained deep learning model that was able to produce a powerful wide-spectrum antibiotic called Halicin [34]. However, one of the most critical shortcomings of AI and a crucial component for achieving revolutionary benefits is the requirement of tons of data for training its models to produce accurate and useful outputs. The combination of AI, IoT, and blockchain technologies can be powerful, where IoT devices provide the data (input), blockchains facilitate their transmission to various machine learning, and deep learning models can translate these data into extremely useful outputs. Some of the newer developments in machine learning are significantly driving blockchains to be better integrated with AI in the health care field. This enables improvements in the security, privacy, functionality, and operational aspects of blockchain technology for health care applications [12,35,36].

### Clinical Scenarios, Potential Applications, and Conclusions

If Chenoa had IoT devices that could monitor her vital signs (particularly temperature) 24/7 and the information was transmitted live via a blockchain to live monitors powered by machine learning algorithms, then her temperature fluctuations and trends would have prompted a return to the transplant center much sooner. Although research on nanotechnology and IoT-based sensors for detecting cancers (or relapses) is in its infancy, IoT devices for monitoring vital signs have already proved their effectiveness in monitoring patient physiology. Moreover, if Chenoa’s EHR was available to all the treating clinicians across the country via a blockchain, it may have triggered a sense of urgency in the local physician treating her.

For Khaled, having his EHR not readily available to a treating emergency room physician is perhaps a classic example of a disjointed medical ecosystem. If there are IoT devices detecting potassium, oxygen, and vital signs in a heart failure patient, they could be instrumental in saving the lives of many patients with heart and kidney diseases. Moreover, having a living will and information on the power of attorney on a blockchain could be very helpful in certain end-of-life cases as well. Finally, for the hundreds of thousands of patients participating in clinical trials, informed consent on a blockchain could be very beneficial for the trial sponsors, ethics boards (eg, institutional review boards), and patient care providers.

Thus, the potential benefits of blockchains are limitless; however, concrete data on long-term clinical outcomes based on blockchains powered and supplemented by AI and IoT are yet to be achieved. Nonetheless, the implementation of blockchains as a novel way to integrate EHRs nationwide and manage common clinical problems in an algorithmic fashion has the potential of saving thousands of lives like those of Chenoa and Khaled.

## Abbreviations

AI: artificial intelligence
EHR: electronic health record
HSMs: hardware security modules
IoT: Internet of Things
